# Vexitoxins: conotoxin-like venom peptides from predatory gastropods of the genus *Vexillum*

**DOI:** 10.1098/rspb.2022.1152

**Published:** 2022-08-10

**Authors:** Ksenia G. Kuznetsova, Sofia S. Zvonareva, Rustam Ziganshin, Elena S. Mekhova, Polina Dgebuadze, Dinh T. H. Yen, Thanh H. T. Nguyen, Sergei A. Moshkovskii, Alexander E. Fedosov

**Affiliations:** ^1^ Federal Research and Clinical Center of Physical-Chemical Medicine, 1a, Malaya Pirogovskaya, Moscow 119435, Russia; ^2^ A.N. Severtsov Institute of Ecology and Evolution, Russian Academy of Sciences, Leninsky prospect, 33, Moscow 119071, Russia; ^3^ Institute of Bioorganic Chemistry, Russian Academy of Sciences, Miklukho-Maklaya street, 16/10, Moscow 117997, Russia; ^4^ Russian-Vietnamese Tropical Research and Technology Center, Coastal Branch, 30 Nguyễn Thiện Thuật, Nha Trang, Vietnam; ^5^ Pirogov Russian National Research Medical University, 1, Ostrovityanova, Moscow 117997, Russia

**Keywords:** Mollusca, Gastropoda, venom evolution, molecular adaptation, ion channel, toxin

## Abstract

Venoms of predatory marine cone snails are intensely studied because of the biomedical applications of the neuropeptides that they contain, termed conotoxins. Meanwhile some gastropod lineages have independently acquired secretory glands strikingly similar to the venom gland of cone snails, suggesting that they possess similar venoms. Here we focus on the most diversified of these clades, the genus *Vexillum*. Based on the analysis of a multi-species proteo-transcriptomic dataset, we show that *Vexillum* species indeed produce complex venoms dominated by highly diversified short cysteine-rich peptides, vexitoxins. Vexitoxins possess the same precursor organization, display overlapping cysteine frameworks and share several common post-translational modifications with conotoxins. Some vexitoxins show sequence similarity to conotoxins and adopt similar domain conformations, including a pharmacologically relevant inhibitory cysteine knot motif. The *Vexillum* envenomation gland (gL) is a notably more recent evolutionary novelty than the conoidean venom gland. Thus, we hypothesize lower divergence between vexitoxin genes, and their ancestral ‘somatic’ counterparts compared to that in conotoxins, and we find support for this hypothesis in the evolution of the vexitoxin cluster V027. We use this example to discuss how future studies on vexitoxins can inform the origin of conotoxins, and how they may help to address outstanding questions in venom evolution.

## 1. Introduction

The order Neogastropoda is a large and successful group of marine gastropod molluscs comprising over 12 000 described species. Most neogastropods are carnivores [1], and many have developed unique biochemical innovations enabling their diverse hunting and defensive strategies [2–5]. The best known of them are venoms of *Conus* snails, comprising structurally diverse neuropeptides, *conotoxins* that cause devastating physiological effects in preys and may be deadly for humans [6]. Due to their ability to selectively block a wide array of ion channels in the nervous system, conotoxins are a major highlight in the natural product-based pharmacology [7,8]. They are typically short (usually not exceeding 40 aa) cysteine-rich peptides, with a high proportion of post-translationally modified residues [9,10]. Conotoxin precursors have a uniform structure, comprising an N-terminal signal sequence, a pro-region and a single mature peptide domain [9,11]. Whereas signal regions are typically highly conserved, the mature peptide domains evolve under strong positive selection and were estimated to be among the fastest evolving animal peptides [12].

Whereas cone snail venoms attract broad interdisciplinary interest, some other yet unstudied biochemically Neogastropoda lineages are likely to have independently evolved similar venoms. Conotoxins are synthesized in a specialized tubular venom gland, an evolutionary innovation of the hyperdiverse superfamily Conoidea [13,14]. It derives from the ancestral glandular dorsal oesophagus fold fused with a mid-gut gland of Leiblein (gL) [15,16], but the superfamily Conoidea is not unique in evolving a secondary secretory gL compartment. The gL was present in the last common ancestor of all Neogastropoda and is present in most extant lineages of the order. The glandular dorsal oesophagus wall, adjoining gL has stripped off to form a novel secretory gland in at least two other lineages independently from Conoidea. Several lines of evidence suggest that each Neogastropoda lineage possessing such derived morphology uses venom to subdue and kill [2,17] or to narcotize the prey [18].

**Figure 1.**
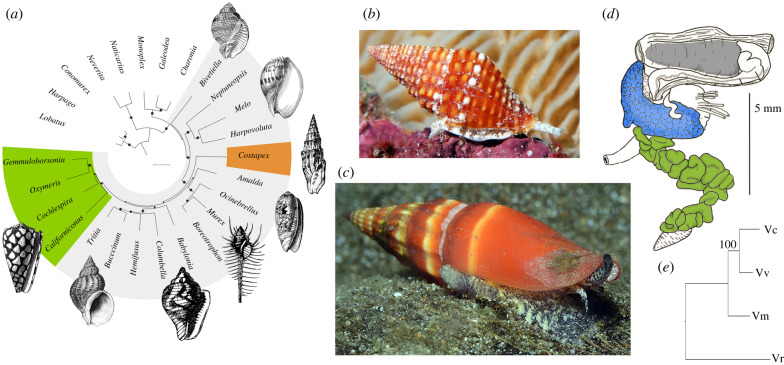
Phylogeny and morphology of *Vexillum*. (*a*) Mitochondrial phylogeny of the Neogastropoda [19]; the family Costellariidae is represented by *Costapex baldwinae*. (*b*) Life specimen of *Vexillum crocatum* (photo courtesy of Janette Johnson). (*c*) Life specimen of *Vexillum coccineum* (photo courtesy of Janette Johnson). (*d*) Foregut anatomy of *Vexillum vulpecula*; sg shown in blue, gL in green. (*e*) Species tree of the four *Vexillum* species analysed herein based on the ML analysis of concatenated aa sequences of 426 BUSCO loci (126 681 aa sites). (Online version in colour.)

In the present study, we focus on the most species rich of these lineages, the genus *Vexillum* (figure 1). The genus comprises a crown radiation of the family Costellariidae [20] and encompasses about 390 living species (Mollusca Base, available at https://www.molluscabase.org/), widely distributed in the shallow water communities of tropical Indo-Pacific. We for the first time demonstrate the existence of venom in *Vexillum*, based on a comprehensive transcriptomic analysis of four species, and support our findings by proteomic profiling. We show that venoms of *Vexillum* are dominated by highly diversified short cysteine-rich peptides that we name *vexitoxins* that in many aspects are very similar to conotoxins. They possess the same precursor organization, display overlapping cysteine frameworks and share several common post-translational modifications (PTMs) with conotoxins. At least five clusters of vexitoxins show detectable structural similarity to conotoxins and are predicted to assume similar domain conformation. Furthermore, we show that multiple unrelated vexitoxins contain the inhibitor cysteine knot (ICK) motif [21,22], a characteristic feature of many pharmacologically relevant animal toxins. For example, ICK defines native structure of δ-, μ-, κ-, ω- conotoxins, the potent blockers of voltage-gated ion channels; among them, the ω- conotoxin MVIIIA was developed into first conotoxin-based analgesic ziconotide (Prialt) approved by FDA [8]. In this context, vexitoxins being highly diversified and sharing key structural features of conotoxins have significant potential to become a novel source of bioactive peptides for drug development and neuroscience research.

## 2. Material and methods

### (a) Specimen collection and tissue sampling

Specimens of four *Vexillum* species, *V. coccineum*, *V. vulpecula*, *V. melongena* and *V. crocatum* var. *cumingi* (figure 1; electronic supplementary material, figure S1) were collected by scuba diving in Nha Trang Bay, Central Vietnam in May 2021. Three former species were sampled on sandy bottom at depths 4–9 m, and *V. crocatum* from a crevice of a reef wall at depth 12 m. Two specimens of each species were dissected for transcriptomic analysis, and three additional specimens of each *V. coccineum* and *V. vulpecula* were dissected for proteomics. Salivary gland (sg) and the tubular gL were preserved individually for each specimen, except *Vexillum crocatum*, for which two sg and two gL were pooled (44VrsggL). Tissues for transcriptomic analysis were preserved in RNA*later* (ThermoFisher) and stored at −20°C. Samples for proteomic analysis were immediately frozen in liquid nitrogen and kept at −70°C.

### (b) RNA extraction and sequencing

RNA was extracted using the standard Trizol method; bioanalyzer traces were used to assess RNA suitability for sequencing. The barcoded libraries for Illumina sequencing were prepared with the Illumina TruSeq RNA sample preparation kit (Illumina, San Diego, USA) following manufacturer's instructions. All libraries were pair-end sequenced on the Illumina HiSeq 4000 (read length 150 bp), at the sequencing facility Genoanalitica (*V. coccineum* and *V. vulpecula*) or at the genomics core facility of Skolkovo Institute of Science and Technology (*V. melongena* and *V. crocatum*).

### (c) Transcriptome assembly and annotation

Raw reads were filtered to remove putative contamination by running FastQ-Screen v. 0.14.1 [23], against 26 genomes of possible laboratory contaminants, either commonly studied model organisms, or taxa that were handled in parallel with our samples during the library preparation. The reads that did not map to any genome were trimmed using Trimmomatic v. 0.39 [24] as reported in [25] and assembled using Trinity v. 2.11 [26] with default parameters. We used RSEM v. 1.3.1 [27] to produce TPM-based measures of transcript abundances, according to the most common practice [25,28,29], and evaluated assemblies completeness by running BUSCO with the metazoan dataset (954 loci) and the Mollusca dataset (5295 loci) [30]. Coding DNA sequences (CDSs) comprising over 35 aa were predicted using ORFfinder and filtered to remove possible cross-contaminations. If a CDS showed a TPM expression level less than or equal to 0.01 relative to an identical CDS from another specimen sequenced at the same facility, the former one was removed. Secreted gene products were identified as CDSs containing a signal sequence identified by SignalP v. 5.0 [31] (*D*-value, *D* ≥ 0.7), but lacking a transmembrane domain, detected by phobius v. 1.01 [32]. The 73 945 CDSs that satisfied these criteria were annotated using BLASTp against the manually curated SWISS-Prot database [33] and by HMMER v. 3.2.1 [34] against the PFam database [35].

Because of the lack of relevant genomic resources, we expected that only a subset of *Vexillum* venom components can be revealed by the reference-based annotation [25]. Therefore, we first performed CDSs clustering, using two alternative approaches: (i) based on the signal sequence identity (PID = 0.65; [25]) detected by CD-Hit [36] and (ii) based on orthologue groups composition inferred by Orthofinder2 [37]. Subsequently, we only focused on those transcript clusters, which included at least one CDSs with the TPM greater than 200. Thus, reduced dataset of 3308 CDSs was manually curated to establish optimal clustering based on (i) signal sequence identity, (ii) orthogroup inference and (iii) reference-based annotation. The CDSs identified as conotoxins, or displaying structural features of conotoxins, were analysed by Conoprec (http://www.conoserver.org/index.php?page=conoprec) to identify domain boundaries, canonical Cys-frameworks and putative PTMs.

To generate additional support for the identified PTMs, we searched for four respective PTMs enzymes, and two enzymes involved in conotoxin folding [38] in the transcriptome assemblies of *V. coccineum* and *V. vulpecula*. All transcripts of *V. coccineum* and *V. vulpecula* that generated a BLASTx hit to the PTM enzymes sequences accessed from SWISS-Prot with aligned length greater than or equal to 50% of the respective database entry, and the *e*-value ≤ 1 × 10−25 were recorded.

The structure modelling was performed using AlphaFold2 [39] implemented in the ColabFold notebook (https://colab.research.google.com/github/sokrypton/ColabFold/blob/main/beta/AlphaFold2_advanced_beta.ipynb). We predicted five models with highest mean pLDDT (Local Distance Difference Test) scores and refined the best scoring model using Amber. The protein structure search was performed by RUPEE [40] (https://ayoubresearch.com/) against the SCOPe database [41].

### (d) Evolutionary analysis

To reconstruct gene tree of the V027 cluster, we codon-aligned 10 complete CDSs of V027 using MACSE v. 2 [42] and performed a ML search by running IQ-Tree with 1000 ultra-fast bootstrap iterations [43] treating three codon positions as separate partitions. We used fixed effects likelihood (FEL), single-likelihood ancestor counting and Fast Unconstrained Bayesian AppRoximation (FUBAR), all implemented in the HyPhy package [44] to test for pervasive selection across the alignment. To infer the sites under episodic diversifying selection, we applied mixed effects model of evolution analysis (MEME).

### (e) Proteomic analysis

The main goal of proteomic analysis was to generate support for the venom components predicted based on the transcriptomic data. Because a notable proportion of these were short peptides, and could be passed to mass-spectrometric analysis without preceding digestion, for each sample, we analysed both, peptidome obtained from the native low-molecular weight peptide fraction and proteome generated from the trypsin-digested proteins greater than 10 kDa (see electronic supplementary material, protocol 1). The peptidome samples were directly subjected to LS-MS/MS analysis following *de novo* sequencing by the PEAKS CMD v. 1.0. We mapped the resulting peptides to the database of all mature peptide sequences of species putative toxins (electronic supplementary material, data S1), accounting for the identical masses of Leu and Ile, and filtered results to keep peptide with at least 80% average confidence. The mass-spectra obtained from the digested samples were searched against the full databases of species CDSs, by running IdentiPy v. 0.3.3.16 [45] followed by Scavager v. 0.2.4 [46], implementing a target-decoy strategy with 1% false-discovery rate cut-off.

## 3. Results

### (a) Transcriptomic proteomic and peptidomic data

Thirteen transcriptomic datasets generated for four species of *Vexillum* (electronic supplementary material, table S1) were similar in terms of the number of reads per sample, and the obtained assemblies were comparable in the BUSCO completeness. The completeness score was consistently slightly lower in sg compared to the gL of the same specimen and slightly lower in *V. melongena* compared to *V. coccineum* and *V. vulpecula*. Furthermore, the proteomic data was obtained for *V. coccineum* and *V. vulpecula*; therefore, we focus on the putative venom components identified in these two species. After the removal of incomplete CDSs and single-CDS clusters, the final dataset included 2187 CDSs allocated to 235 putative clusters. Of these, 850 and 817 CDSs represented putative venom components of *Vexillum coccineum* and *V. vulpecula*, respectively.

The largest number of unique matches (727) in proteome was obtained from the specimens of *V. coccineum* gL, and the lowest (543 matches) from *V. vulpecula* sg (electronic supplementary material, table S2 and figure S2). These generated support for 439 and 322 CDS, respectively; however, most supported CDS correspond to non-unique matches, because most peptides have generated hits to multiple database entries, which we collectively refer to as ‘protein group’. When carboxylated glutamic acid and hydroxy-prolyne were set as variable modifications, additional sets of peptides have matched, again with larger numbers of hits in gL. Finally, from 137 to 262, native peptides per tissue-species series were identified by *de novo* peptide sequencing. The largest (479) and the smallest (349) total numbers of supported CDS corresponded to gL of *V. coccineum* and sg of *V. vulpecula*, respectively. The lack of the support for the remaining CDSs may be explained methodologically, by the inherent difference in resolution between the RNA-Seq and shotgun proteomics, but as well, by some biological reasons, such as the more dynamic proteome composition, related e.g. to regular venom discharges. We aligned all the detected peptides to the matching query CDSs (electronic supplementary material, data S1 and S2) and calculated the number of predicted mature peptide aa residues of each CDS, supported by the detected peptides. This value was divided by the length of the mature region, and the resulting ratio was used as a measure of support; below we report it for three best-supported CDSs of each putative toxin cluster inferred from the transcriptomic data.

### (b) Major groups of salivary gland and gland of Leiblein transcripts are related to functions of venom

**Figure 2.**
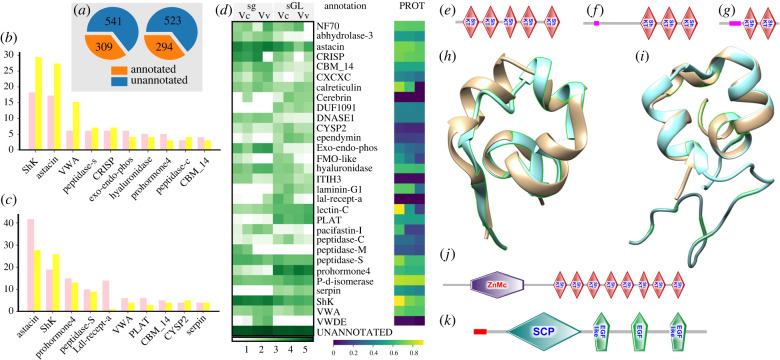
Major classes of the annotated *Vexillum* transcripts. (*a*) Proportions of annotated and unannotated transcripts in *Vexillum coccineum* (left) and *V. vulpecula* (right). (*b*,*c*) Ten most diversified classes of annotated transcripts in (*b*) salivary gland (sg) and (*c*) gland of Leiblein (gL); pink (left)—*Vexillum coccineum*, yellow (right)—*V. vulpecula*. (*d*) Heatmap of log10-transformed summed TPM expression levels of 30 most highly expressed annotated transcript classes per dataset. On the right heatmap of the cluster support in proteomic data: three cells in a horizontal row correspond to three CDS of a cluster best represented in our proteomic data. (*e*–*g*) SMART structure of multi-ShK transcripts: (*e*) Vc0003648, (*f*) Vc0000412 and (*g*) Vc0000028. (*h*) Superposition of the ShKT-like domain Vc0000412-1 (beige) and *Stichodactyla helianthus* K channel toxin (d1beia). (*i*) Superposition of the ShKT-like domain Vv0001739-1 (beige) and crisp-3 member Natrin of *Naja atra* (d2giza2). (*j*,*k*) SMART structures of highly expressed multi-domain sg transcripts: (*j*) astacin Vv0000203, 14Vvsg:TPM-16763.81; (*k*) CAP Vc0000374 8Vcsg: TPM-4360.1, 9Vcsg:TPM-5528.32. (Online version in colour.)

Confident BLAST or HMMER hits were obtained for 309 and 294 CDSs of *V. coccineum* and *V. vulpecula*, respectively, or 36.4% and 36.0% of the putative venom components in the two species, respectively (figure 2*a*), and represent 47 Pfam gene families. Proteins bearing *Stichodactyla heliantus* K channel toxin-like domains (ShKT-like) and metalloproteases, mainly of astacin type were the most diversified of annotated clusters in both, the sg and the gL of both species (figure 2*b–d*). We identified 98 complete transcripts of ShKT-like domain-bearing proteins [47]. Most of them comprise two to six ShKT-like domains (figure 2*e*–*g*), and can be classified as multi-ShKT-like peptides [47]. The predicted ShKT-like domains typically show a characteristic arrangement of six cysteine residues and, in most cases, would assume a conformation, closely matching that of *Stichodactyla heliantus* K channel toxin (figure 2*h*). Nevertheless, the predicted folding of a few domains, identified as ShKT-like by HMMER, shows closer match to some snake venom peptides, such as natrin (figure 2*i*). Both the ShK toxin and natrin are potent ion channel blockers, and it remains to be established, whether the structurally similar peptides of *Vexillum* also target ion channels. Furthermore 10 revealed clusters of transcripts, bearing an M12 metalloprotease domain, all have encoded ShKT-like domains C-terminally, their number ranging from 2 to 14 (figure 2*j*).

Besides metalloproteases, other classes of putative cyto- or haemolytic compounds showed high expression levels, including transcripts with referred to abhydrolase family, peptidases –S, –M and –C, lectin-C, chitinase (CBM_14). Of these, abhydrolase, M12 metalloproteases and various peptidases are common in venoms of snakes and insects, and lectins are highly expressed in distantly related to cone snails of the genus *Clavus*, being possibly an adaptation counterbalancing the lower compared to cone snails efficiency of venom delivery [48].

Among the putative venom components similar with non-conotoxin components of *Conus* venoms, we revealed diversified and highly expressed in the gL transcripts, provisionally annotated as prohormone-4, CRISP and hyaluronidases, the latter two mainly expressed in sg (figure 2*k*). Interestingly, the serine-type protease inhibitors in *Vexillum* are on the contrary very different from those found in *Conus*. While kinitz and kazal types are common in *Conus* venom, those detected in *Vexillum* showed high identity to pacifastins and serpins, the former class mainly known from arthropods, the latter commonly found in venoms of snakes, spiders and hymenopterans.

Broad representation and high expression of the peptide classes with a well-established connection to venom functions further reconfirm the relevance of both the sg and gL to envenomation. Although in-depth characterization of the annotated clusters is worthwhile, below we focus on the highly diversified putative venom peptides with structural properties of neurotoxins, exploring their structural similarities with conotoxins.

### (c) Conotoxin-like peptides

Most-predicted-secreted CDSs could not be annotated using reference databases. Here, we consider them together with the total of 32 CDSs that showed structure similarity with conotoxins, because a large set of the unannotated CDSs appears to share characteristic features of conotoxins. These features are: (i) the canonical precursor structure with a conserved signal sequence, and a rather short, variable mature domain represented by a single copy; (ii) a high number of cysteine residues in the mature domain that form distinctive Cys-frameworks; and (iii) a high number of post-translationally modified residues in the mature region.

**Figure 3.**
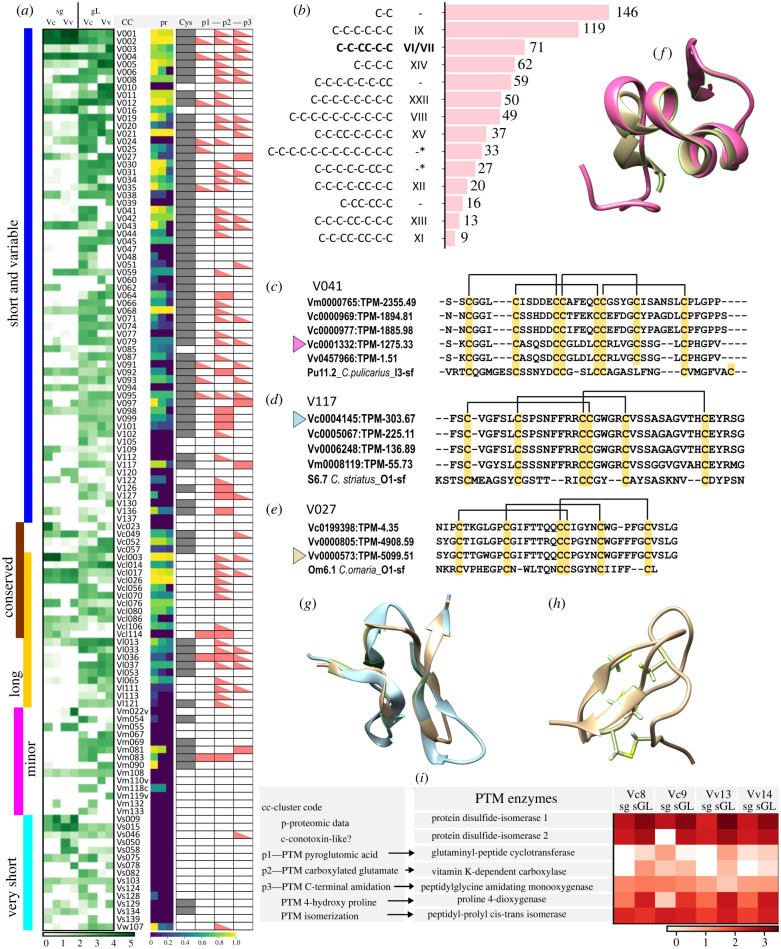
Expression and structural features of the unannotated *Vexillum* transcript clusters. (*a*) Heatmap of log10-transformed expression of 118 unannotated transcripts clusters in sg and gL transcriptomes of *Vexillum coccineum* and *V. vulpecula*. Column pr—support of clusters in proteomic data (markup like in figure 2). Column Cys: grey marks the presence of a conserved Cys-framework across most cluster's, sequences. Columns p1–p3—prediction of three PTMs common in conotoxins: p1—N-terminal pyroglutamate, p2—carboxy-glutamate, p3—C-terminal amidation. (*b*) Most common Cys-frameworks in unannotated clusters of putative *Vexillum* toxins. (*c*–*e*) Mature peptide alignment in three clusters of vexitoxins with closest conotoxin matches; (*c*) Cluster V041, (*d*) V117 and (*e*) V027. (*f*) Superposition of the vexitoxin Vc0001332 versus ρ-conotoxin TIA. (*g*) Superposition of the vexitoxin Vc0004145 versus conotoxin GS. (*h*) three-dimensional structure of the vexitoxin Vv0000573. (*i*) Heatmap of log10-transformed expression levels of seven key PTM enzymes in *Vexillum* *coccineum and V. vulpecula*. (Online version in colour.)

The 1580 unannotated-secreted CDSs were classified into 146 clusters; each cluster was assigned a code based on (i) its summed expression (electronic supplementary material, table S3), (ii) CDS length and (iii) degree of variation within a cluster. In total, 117 clusters demonstrated high expression in at least one of the profiled specimens (TPM ≥ 1000) or moderately high expression (TPM ≥ 200) across several specimens. Their records in the heatmap (figure 3*a*) are grouped based on the length of the included CDSs, and the degree of sequence variation within a cluster. It can be noted that the clusters of medium-sized CDSs (precursor length ranging from 40 to 200 aa), with over 10% variable aa sites are much broader represented in gL than in sg. Those clusters, where transcripts mature regions comprise at least two cysteine residues, and share the same or compatible Cys-frameworks are highlighted in grey in the column ‘Cys’ (figure 3*a*). Of 942 complete transcripts in these clusters, 445 (or almost half) encode mature toxins with canonical Cys-frameworks known from conotoxins. Of the 14 most common frameworks (shared by no less than 10 predicted CDSs), nine are known in conotoxins (figure 3*b*). For example, the framework IX found in 119 *Vexillum* CDSs is present in most P-superfamily conotoxins [10], and the framework VI/VII, known as the ICK [10], is common in the O-, H- and N- conotoxin superfamilies. This framework shared by 71 putative vexitoxins is the third most common in *Vexillum*. The O1-superfamily conotoxins with the framework VI/VII are potent blockers of voltage-gated Na^+^ channels (pharmacological families *δ*- and μ-), K^+^ channels (*κ*-) and Ca^2+^ channels (*ω*-) [8,10]. The molecular targets of the remarkably diversified framework VI/VII *Vexillum* venom peptides are still to be determined, and it is tempting to speculate that functionally they may play a similar role to that of the O1-superfamily conotoxins.

The CDS Vc0001332 (cluster V041) has an uncommon Cys-framework XI with four disulfide bounds. While its predicted sequence is closest to that of *Conus pulicarius* Pu11.2 (figure 3*c*), the predicted structure shows highest similarity to the much shorter ρ-conotoxin TIA (A-superfamily) of the fish-hunting species *Conus tulipa* (figure 3*f*). The putative *Vexillum* toxins Vc0004145 and Vv0000573 both contain an ICK motif with its signature connectivity 1–4, 2–5, 3–6 (figure 3*g,h*) and show closest sequence similarity to *Conus striatus* S6.7 and *Conus omaria* Om6.1, respectively (both O1 superfamily). The modelled three-dimensional structure of the Vc0004145 showed a close match to the synthetic μ-conotoxin GS of *Conus geographus* (figure 3*g*).

To estimate whether the predicted *Vexillum* toxins bear same PTMs as conotoxins, we summarized the PTM predictions obtained from ConoPrec (figure 3*a*, columns p1–p3) and corroborated these by the expression data of the corresponding PTM enzymes in the sg and gL transcriptomes (figure 3*i*). Our results suggest that these PTMs are likely to be quite common in *Vexillum* toxins. Among the predicted PTMs, the gamma-carboxylated glutamate was most common (395 putative toxins from 56 clusters); however, the reliability of this PTM prediction is questionable [49]. By setting glutamate carboxylation as a variable modification, we recovered 25 to 104 additional unique monoisotopic masses per sample, with larger number of additional hits in the query gL CDSs (electronic supplementary material, table S3), supporting presence and higher frequency of this PTM in the gL toxins. The C-terminal amidation was predicted as the second most common PTM (236 putative toxins from 32 clusters). Furthermore, we detected seven essential PTM enzymes in the analysed transcriptomes (figure 3*i*). Protein disulfide isomerase, prolyl 4-hydroxylase and peptidyl-prolyl cis-trans isomerase showed highest overall expression levels and higher expression in the gL. Glutaminyl-peptide cyclotransferase, Vitamin K-dependent carboxylase and peptidyl-glycine amidating monooxygenase were detected in all gL transcriptomes, but the former two were lacking in two sg datasets.

### (d) Cross-tissue recruitment exemplified by the V027 cluster evolution

**Figure 4.**
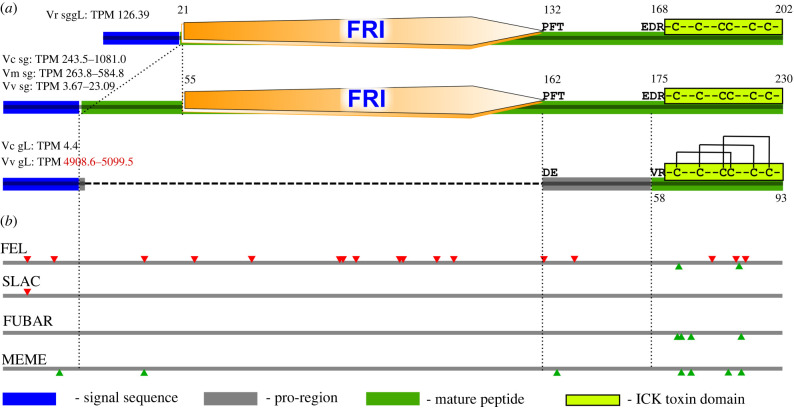
Precursor structure and evolution of the cluster V027 sequences in *Vexillum*. (*a*) Precursor structures in V027. Top—long orthogroup, Vr0003450; middle—long orthogroup, Vc0001640; bottom—short orthogroup, Vv0000573. (*b*) Codons under negative selection (red, above grey lines), pervasive positive selection or episodic diversifying selection (green, below grey lines). (Online version in colour.)

The cluster V027 sequences appeared to represent two orthogroups with considerable differences in sequence length and tissue expression (figure 4). The long orthogroup sequences are about 230 aa long and are expressed in salivary glands of *Vexillum coccineum* and *V. melongena*. The short orthogroup sequences are 92–93 aa long and show very low expression in gL of *Vexillum coccineum* and very high expression in *V. vulpecula* gL (TPM approx. 5000). Both orthogroups share a high-identity N-terminal signal sequence, and a short (35 aa) C-terminal fragment bearing an ICK motif. The observed length difference is due to the presence in the long orthogroup sequences of a conserved 111 aa long region annotated by HMMER as Frizzled domain. The long orthogroups sequences are predicted to cleave into two fragments, an N-terminal signal sequence and a long C-terminal mature peptide combining the Fz-domain with its flanking regions including the ICK-bearing domain. On the contrary, the short orthogroup sequences cleave in a manner similar to conotoxin precursors: into (i) a signal sequence, (ii) a short pro-region and (iii) a short mature peptide, exactly corresponding to the C-terminal ICK-bearing domain. Its sequence shows highest similarity to the omega-conotoxin Om6.1 of *Conus omaria* (figure 3*e*) and adopts a conformation characteristic for omega toxins (figure 3*e*). Three monoisotopic masses uniquely matching the Frizzled domain sequence are detected in proteome of *V. coccineum* (present in all six analysed species-tissue series), but are lacking in *V. vulpecula*. Conversely, peptides matching the V027 ICK motif were only detected in the gL samples of *Vexillum vulpecula*.

The reconstructed cluster V027 phylogeny (electronic supplementary material, figure S3) and the orthogroups distribution across species suggest that the longer orthogroup transcript structure is ancestral, and the short orthogroup structure is derived. Our selection analysis identified 17 alignment sites under negative selection, predominantly in the Fz domain. Conversely, of eight alignment sites subject to pervasive positive selection (FEL, SNAP and FUBAR), or evolving under diversifying selection (MEME), five are within the ICK-bearing domain.

This pattern is consistent with the second orthogroup descending from the first one resulting from a gene duplication event that has occurred before the split of *V. coccineum* and *V. vulpecula*. Following the gene duplication, the second orthogroup sequences lost the Fz-domain and acquired a cleavage site at the N-terminal boundary of the ICK domain. The shortened mature peptide region was rapidly evolving under positive selection and gained high tissue-specific expression in gL of *Vexillum vulpecula*. The very high expression of the transcripts Vv0000573 and Vv0000805 in *V. vulpecula* gL, the presence of the matching translation products in the proteome and their three-dimensional structure determined by the ICK, all point at the relevance of this cluster to envenomation.

## 4. Discussion

### (a) Vexillum toxins are a novel source of bioactive neuropeptides

The molecular targets of conotoxins, a wide array of ion channels and receptors in the nervous system, have made them a promising source of analgesics and a potentially preferable treatment for long-term pain management [8]. The relevance of conotoxins as pharmacological agents can be explained by the fact that venoms in some cone snail species were evolved specifically to subdue vertebrate preys [2]. Therefore, the fish-hunting *Conus* species (or more broadly, those venomous lineages specialized to hunt vertebrate preys) are the first priority for drug discovery. While this logic formulates a ‘pragmatic’ approach to prioritizing targets of resource-consuming drug development, it would *a priori* eliminate many potentially valuable candidate molecules. For example, the α-conotoxin Rg1a, an inhibitor of the *α*9*α*10 nicotinic acetyl-choline receptors, proved to be a potent analgesic [50], despite being produced by a worm-hunter species *Conus regius*. Similarly, sea anemones do not feed on vertebrate preys; nevertheless, ShK toxins have high affinity to the vertebrate subtypes of potassium channels [51]. These examples may be explained by either broad taxonomic distribution of relevant molecular targets, or by the existence of defensive components of venoms, targeted to vertebrate predators. The defensive venoms targeted to vertebrates may have a much broader distribution across animal lineages, compared to the predatory toxins targeted to vertebrates. In this context, a broader sampling of venomous animal taxa is crucial to systematically explore their molecular adaptations to both hunting and defense, and to efficiently reveal novel bioactive compounds.

In the present study, we make a first step towards documenting venom composition of a highly diversified, yet previously unexplored lineage of venomous gastropods, the genus *Vexillum*. Due to the small size of its glands compared to a venom gland of cone snails, collection of sufficient material for bioassays of *Vexillum* venoms is a challenging task. To overcome this challenge, we used RNA-Seq and shotgun proteomics approaches that both require little material but enable a comprehensive analysis of venom composition. We uncover highly diversified short secreted peptides referable to CRISP neuropeptides class in both, the salivary gland and specialized tubular gland of Leiblein of *Vexillum*. We analyse the vast diversity of short Cys-rich secreted peptides of *Vexillum* in the context of their similarity with conotoxins. In total, 55 transcript clusters (47 supported by proteomic data) show structural features characteristic of conotoxins: short mature domain, largely conserved Cys-framework and the presence of some signature PTMs. Although we do not have functional data to support pharmacological activity of the respective gene products, we present strong evidence that (i) their translation products do exist in the protein fraction of the analysed secretory glands and (ii) a sizeable fraction of them possess structural features characteristic of toxins. It is thus logical to propose that *Vexillum* toxins target same physiological circuits of preys and predators as do the conotoxins.

### (b) Comparative framework for venom evolution inference in Neogastropoda

Venoms have emerged over a hundred times in independent metazoan lineages [52], offering a unique opportunity for studying genetic underpinnings of repeated key traits apparition [53,54]. Being a key adaptation for predation and defense, venoms to a great extent affect species fitness and biology [55,56]. Setting up venom production requires novel specialized tissues and glands, in which a set of genes originally not related to the venomous function is recruited and modified to encode potent toxins. Most animal toxins represent rather few broad classes of proteins [54], but being broadly distributed across unrelated venomous animal taxa, they expectedly have been recruited from very different genomic backgrounds [57]. This general trend to convergent evolution provides a unique opportunity to disentangle the interplay of conserved and lineage-specific mechanisms that govern recruitment and evolution of venom peptides. To enable such inference globally, a scalable comparative framework should be generated to cover entire phylogenetic diversity of venomous animals. Notwithstanding, taxonomically restricted fragments of such framework may yield deep insights into genomic underpinnings of evolution and regulation of venomous function. Currently, most efforts to this end focus on the well-characterized taxa of venomous animals, mainly on snakes (e.g. [57,58]), and extending such studies to new system(s) will greatly magnify the power of comparative analysis. Essentially such system can be seeded by a pair of distantly related taxa that have independently acquired venoms. Cone snails and *Vexillum* represent independent evolutionary successful radiations of venomous neogastropods with convergent transformations of foregut enabling venom production and therefore constitute a perfect system.

Evolutionary histories and distributions of *Conus* and *Vexillum* display multiple parallels. Similar to *Conus*, *Vexillum* is species rich and forms a crown group of its respective family, the Costellariidae [20,59]. Similar to *Conus*, *Vexillum* underwent a major diversification in the Miocene [60], and its present-day diversity is associated with tropical shallow waters of Indo-Pacific. Therefore, the adaptive radiations of *Conus* and *Vexillum* were probably shaped by the same set of factors, and acquisition of venom likely has played a major role in the success of both taxa. Within this system, repeated recruitments of a novel specialized secretory tissue of gL allow comparative analysis of the genome evolution processes underpinning emergence of venom gene superfamilies, and establishment of their regulatory pathways. Because tubular gL has the same developmental origin in *Vexillum* and *Conus* (as stripped off dorsal oesophagus wall), the gene expression patterns in the ancestral tissues were presumably closely comparable among them. Conversely, sg is homologous *and* morphologically conserved across Neogastropoda, and also produces some classes of bioactive compounds in both cone snails and *Vexillum* [3,61]. This two-tissue system enables a comparative analysis of modes and tempos of molecular evolution, as well, as investigation of cross-tissue gene superfamilies recruitment between sg and gL. In the present study, we demonstrate an example of the sg–gL cross-tissue recruitment in the *Vexillum* V027 cluster.

Our results suggest that after an initial gene duplication, the gene structure of the new paralogue was modified to produce a short ICK-bearing toxin. Interestingly, the mounting expression of this toxin in gL of *V. vulpecula* was accompanied by the reduction of the ancestral (long) paralogue expression in the sg, suggesting that the functionality of their gene products may to some extent be complementary or overlap. What we find remarkable in this example is that we were still able to capture the low-expression counterpart of the ancestral (long) orthogroup in the sg of *V. vulpecula*. Furthermore, we detected a low-expression ‘prototype’ of the ICK-bearing toxin gene (the short orthgroup) in the gL of *V. coccineum*, where it is expressed alongside the ancestral orthogroup, but with an order of magnitude lower expression level.

The observed distribution of orthogroups across tissues and species of *Vexillum*, as well, as the distribution of ShKT-bearing transcripts, suggests that there remains some functional overlap between the sg and gL in *Vexillum*, in relation to envenomation. If true, such overlap may generate a ‘highway’ for cross-tissue recruitment of venom components in the evolutionary young gL [20,62]. Therefore, a sizeable fraction of venom components in *Vexillum* is likely to result from recent recruitment events, and so, despite the inherent quick divergence from the ancestral state, the structural or sequence similarity of these venom genes with their non-venomous paralogues may still be traceable. If true, *Vexillum* venoms may become an ideal system to study origin and early evolution of venomous function in general. Particularly, outcomes of this analysis have a great potential to inform the evolution of conotoxins. Genomic source of conotoxin genes remains unknown, mainly because these genes evolve too fast, and the venomous function has originated in the ancestors of cone snails too long ago [14] for detection of the toxin genes ancestry to be possible. However, because *Vexillum* and *Conus* share a common ancestor within the Neogastropoda, their genomic background is largely the same. Therefore, venom evolution inference in *Vexillum* will give a shortcut to identifying the set of ancestral Neogastropoda genes amenable for venom function, and this knowledge, in turn, will generate sensible hypotheses on the evolutionary origin of conotoxins.

## Data Availability

The transcriptomic sequencing data are deposited under the Bioproject PRJNA797643. Raw proteomic data are available in the ProteomeXchange(PXD031020) and MassIVE [ftp://MSV000088687@massive.ucsd.edu]. Sequences of the predicted *Vexillum* toxins are provided in the electronic supplementary material, data files. The essential Python scripts are available at https://github.com/SashaFedosov/Vexillum/). The data are provided in the electronic supplementary material [63].
